# The first steps in vision: cell types, circuits, and repair

**DOI:** 10.15252/emmm.201810218

**Published:** 2019-01-22

**Authors:** Botond Roska

**Affiliations:** ^1^ Institute of Molecular and Clinical Ophthalmology Basel Basel Switzerland; ^2^ University of Basel Basel Switzerland; ^3^ Friedrich Miescher Institute Basel Switzerland

**Keywords:** cell type‐specific gene therapy, FRMD7, neuronal circuits, retinal disorders, vision, Neuroscience

## Abstract

Dysfunction of the key sense of vision, leading to visual handicap or blindness, has a crucial effect on day‐to‐day life. In this commentary, I will summarize the work in my laboratory that is focused on a basic understanding of visual processing and the use of this information to understand disease mechanism and to develop correcting therapies. We are beginning to understand how cell types of the visual system interact in local circuits and compute visual information. This has brought insight into mechanisms of cell‐type‐specific diseases and has allowed us to design new therapies for restoring vision in genetic forms of blindness.

## Cell types, circuits, and computations

We were intrigued to learn how cell types and circuits of the visual system extract features from the visual scene. We started our investigations by creating an atlas of cell‐type transcriptomes in the mouse retina (Siegert *et al*, 2012) and found that each adult retinal cell type expresses a specific set of genes, including a unique set of transcription factors. This novel resource has allowed us to manipulate retinal cell types using mouse genetics or viruses and to explore the logic by which visual circuits extract information from the visual scene.

### Multifunctional cell types in the retina

The first insight we gained was that retinal cell types and circuits are multifunctional and can therefore perform radically different functions depending on the visual input or even when the visual input is the same. Over time, we have reported several examples of such circuits.

The first case was a retinal circuit that specializes in the detection of approaching objects, such as looming predators (Münch *et al*, 2009). Together with our collaborator Rava Azaredo da Silveira, we identified an approach‐sensitive ganglion cell type in the mouse retina, resolved elements of its afferent neural circuit, and described how these confer approach sensitivity to the ganglion cell. The essential building block of the circuit is a rapid inhibitory pathway that selectively suppresses responses to non‐approaching objects. This rapid inhibitory pathway was described previously in the context of night‐time vision. In day‐time conditions, the same pathway conveys signals in the reverse direction. This demonstration of the dual activity of a neural pathway related to different physiological conditions illustrated the efficiency with which several functions can be accommodated in a single circuit.

The second example was a retinal circuit that switches function depending on the ambient illumination (Farrow *et al*, 2013). By sliding through light levels from starlight to daylight, we identified retinal ganglion cell types that abruptly and reversibly switch the weighting of center and surround interactions in their receptive field around cone threshold. Two‐photon‐targeted recordings together with genetic and viral‐tracing experiments identified the circuit element responsible for the switch as a large inhibitory neuron that acts directly on ganglion cells. The experiments suggested that weak excitatory input via electrical synapses, together with the spiking threshold in inhibitory cells, acts as a switch. This work demonstrated that circuits in the retina can quickly and reversibly switch between two distinct states, implementing distinct perceptual regimes at different light levels. Furthermore, it revealed a switch‐like component in the spatial integration properties of human vision at cone threshold.

The third example was based on the discovery that rods, which act as photoreceptors in nightlight, switch their function in daylight (Szikra *et al*, 2014). Vertebrate vision relies on two types of photoreceptors, the rods and the cones, that signal increments in light intensity with graded hyperpolarizations. Rods operate in the lower range of light intensities and cones at brighter intensities. The receptive fields of both photoreceptors exhibit antagonistic center‐surround organization. We demonstrated that mouse rods at bright‐light levels act as relay cells for cone‐driven surround inhibition. Thus, when they are not directly sensing light, rods are not left without a task: They join the cone circuit.

The fourth example was a circuit that can have different functions even when the visual input is the same (Drinnenberg *et al*, 2018). We chemogenetically perturbed horizontal cells, which are an interneuron type providing feedback at the first visual synapse while monitoring light‐driven spiking activity in thousands of ganglion cells, the retinal output neurons. We uncovered six reversible perturbation‐induced effects in the response dynamics and response ranges of ganglion cells. A computational model of the retinal circuitry reproduced all perturbation‐induced effects and led us to assign specific functions to horizontal cells with respect to different ganglion cell types. Our combined experimental and theoretical work revealed how a single interneuron type can differentially shape the dynamical properties of distinct output channels of the retina.

### Computations from retina to cortex

The second insight we generated was the combination of visual information from several visual channels by cells of the lateral geniculate nucleus (LGN) and primary visual cortex and the resulting computation of new visual features.

First, we determined different modes of visual integration in the LGN (Rompani *et al*, 2017). The thalamus receives sensory input from different circuits in the periphery, but how these sensory channels are integrated at the level of single thalamic cells was not well understood. We performed targeted single‐cell‐initiated transsynaptic tracing to label the retinal ganglion cells that provide input to individual principal cells in the mouse LGN. We identified three modes of sensory integration by single LGN cells. In the first, few cells of mostly the same type converged from one eye, indicating a relay mode. In the second, many ganglion cells of different types converged from one eye, revealing a combination mode. In the third, many ganglion cells converged from both eyes, revealing a binocular combination mode in which functionally specialized ipsilateral inputs joined broadly distributed contralateral inputs. Thus, the LGN employs at least three modes of visual input integration, each exhibiting different degrees of specialization.

Second, we provided causal evidence for retina‐dependent and retina‐independent visual motion computations in primary visual cortex (Hillier *et al*, 2017). How neuronal computations in the sensory periphery contribute to computations in the cortex was not well understood. We examined this question in the context of visual motion processing in the retina and primary visual cortex. We genetically disrupted retinal direction selectivity, either along only the horizontal axis using FRMD7 mutant mice or along both cardinal axes using starburst cell‐ablated mice, and monitored neuronal activity in layer 2/3 of primary visual cortex during visual motion. In control mice, we found a strong direction bias for posterior visual motion, which occurs naturally when the animal moves forward. In mice with disrupted retinal direction selectivity, the proportion of posterior motion‐preferring cells decreased significantly and their speed tuning changed. Thus, functionally distinct, retinal direction selectivity‐dependent and selectivity‐independent computation of visual motion occurs in the cortex.

Third, we showed that the visual cortex of mice is organized in layer‐specific cortical network modules (Wertz *et al*, 2015). We used single‐cell‐initiated, monosynaptically restricted retrograde transsynaptic tracing with rabies viruses expressing GCaMP6s to image the visual motion‐evoked activity of individual layer 2/3 pyramidal neurons and their presynaptic networks across layers in mouse primary visual cortex. Neurons within each layer exhibited similar motion direction preferences, forming layer‐specific functional modules. In one‐third of the networks, the layer modules were locked to the direction preference of the postsynaptic neuron, whereas for other networks, the direction preference varied by layer. Thus, this work revealed the existence of feature‐locked and feature‐variant cortical networks.

## A cell‐type‐specific disease mechanism

We designed an experimental logic to identify the relevant cell types and circuits of genetic diseases of vision with unknown pathology. First, we focused on the function of a specific retinal circuit and proceeded to study how cell types participate in a given computation. Then, we revealed the gene expression patterns of the relevant cell types and linked cell‐type‐specific genes to human monogenic diseases. Finally, we connected the symptoms of human diseases to the identified cell types and circuits.

Such an approach has led us to identify the circuit mechanism in a common human neurodevelopmental disease: FRMD7 gene‐associated congenital nystagmus. We linked a key symptom, loss of the optokinetic response, to a single retinal cell type (starburst cells) and a retinal computation, i.e., the computation of motion direction (Yonehara *et al*, 2016).

We developed this insight by following the experimental logic outlined above.

We first investigated the development of the circuit involved in the computation of direction selectivity (Yonehara *et al*, 2011). We followed the spatial distribution of synaptic strengths between starburst and direction‐selective ganglion cells during early postnatal development before these neurons can respond to a light stimulus. We showed that an asymmetry develops rapidly over a 2‐day period through an intermediate state in which random or symmetric synaptic connections have been established. The development of asymmetry involved the spatially selective reorganization of inhibitory synaptic inputs. This work demonstrated a rapid developmental switch from a symmetric to asymmetric input distribution for inhibition in the neural circuit of a principal cell.

Next, we identified the key synapse responsible for the computation of motion direction (Yonehara *et al*, 2013). Using the gene expression atlas developed in our laboratory, we found that FRMD7 was specifically expressed in starburst cells (Yonehara *et al*, 2016). We showed that mutation of FRMD7, a gene that is defective in human congenital nystagmus, leads to selective loss of the horizontal optokinetic reflex in mice, as it does in humans. Together with our collaborator Andreas Hierlemann, we found that this was accompanied by selective loss of horizontal direction selectivity in retinal ganglion cells and the transition from asymmetric to symmetric inhibitory input to horizontal direction‐selective ganglion cells. This work identified FRMD7 as a key regulator in the development of neuronal circuit asymmetry and suggested the involvement of a specific inhibitory neuron type in the pathophysiology of a neurological disease.

Finally, we developed functional ultrasound imaging to record whole‐brain activity in behaving mice at a resolution of ~ 100 μm and compared activity in healthy and FRMD7 mutant mice (Macé *et al*, 2018). In healthy mice, we detected 87 active brain regions during visual stimulation that evoked the optokinetic reflex. Using FRMD7 mutant mice, we identified a subset of regions whose activity was reflex‐dependent. Our work identified the brain regions affected in a mouse model of congenital nystagmus and provided an experimental approach to monitor whole‐brain activity of mice in normal and disease states.

## Cell‐type‐targeted repair

We used our understanding of the activity, connectivity, and gene expression pattern of retinal cell types to design cell‐type‐targeted optogenetic therapies for blinding diseases. We concentrated on a group of genetic diseases, termed retinitis pigmentosa, in which blindness is caused by photoreceptors losing light sensitivity. We showed proof of principle for making key retina cell types light sensitive using cell‐type‐targeted optogenetic approaches (Lagali *et al*, 2008; Busskamp *et al*, 2010). We restored visual function first in animal models of retinitis pigmentosa. With our collaborators Jose‐Alain Sahel and Serge Picaud, we then provided proof of concept for visual restoration in human retinas *ex vivo* and identified blind patients who could benefit from the potential therapy. We are currently working on translation of the therapy to patients: A first phase clinical trial run by GenSight Biologics Inc. is ongoing.

A further therapeutic approach to photoreceptor‐based blindness is to prevent the loss of photosensitivity. Working toward this goal, together with our collaborator Witold Filipowicz, we identified a key microRNA‐based pathway involved in the maintenance of the photosensitive subcellular compartment, the outer segment of photoreceptors (Busskamp *et al*, 2014). We then used the identified molecules to grow outer segments and enable photosensitivity in embryonic stem cell‐derived retinoids *in vitro*. We are currently experimenting with these molecules to prevent loss of photosensitivity in retinitis pigmentosa.

A key limitation in gene therapy has been the lack of cell‐type‐specific gene delivery vectors. Adeno‐associated viral vectors (AAVs) are frequently used for gene delivery, but targeting expression to specific cell types has been a challenge. We created a library of 230 AAVs, each with a different synthetic promoter designed using four independent strategies (preprint: Juettner *et al*, 2018). We showed that ~ 11% of these AAVs specifically target expression to neuronal and glial cell types in the mouse retina, the mouse brain, the non‐human primate retina *in vivo*, and the human retina *in vitro*. We demonstrated applications for recording, stimulation, and molecular characterization, as well as the intersectional and combinatorial labeling of cell types. These resources and approaches allow economic, fast, and efficient cell‐type targeting in a variety of species, both for basic science and for gene therapy.

An additional limitation of current gene therapy approaches is that once injected into the body, viral vectors can no longer be controlled. Together with our collaborator Daniel Mueller, we have begun to address this problem. We have bound viruses to magnetic nanoparticles and showed that these particles can be remote controlled in the brain using a magnetic field in a way that leads to infection; we named the process “virus stamping” (Schubert *et al*, 2018).

## Summary

For a long time, studies of neuronal circuits and of visual diseases have been pursued separately. In recent years, the concept of cell types (structurally, functionally, and transcriptomically similar groups of cells) has brought the two fields together. Cell types are the basic building blocks of neuronal circuits, and technologies to deliver genes to cell types are now core components of neuronal circuit studies. Since most retinal diseases are cell‐type‐specific, the notion of cell types and cell‐type‐targeted gene delivery is also at the center of research on disease mechanisms and therapy. As both fields mature, the concepts and tools developed in basic circuit science will help in the development of therapy and, conversely, the concepts and tools being developed for therapy will open the door to increased sophistication in our understanding of the structure and function of neuronal circuits.

## A personal note

I enjoy thinking and exploring. I was lucky that over the last 13 years I received extraordinary generous support for these activities from Novartis and from the Friedrich Miescher Institute (FMI), for which I am deeply grateful. Currently, I am occupied by thinking about and exploring how can we understand the biology of humans and to develop therapy. Toward this direction—with the generous support of Novartis, the University of Basel, the University Hospital of Basel, and the city of Basel—and together with my colleague and friend Hendrik Scholl, we started a new institute, the Institute of Molecular and Clinical Ophthalmology Basel (IOB). I feel similarly as I felt at the time when I started my laboratory in 1995: a bit insecure, rejuvenated, and full of plans.

## Conflict of interest

Botond Roska is a consultant for Novartis.
